# Interesterified Fats Induce Deleterious Effects on Adipose Tissue and Liver in LDLr-KO Mice

**DOI:** 10.3390/nu11020466

**Published:** 2019-02-22

**Authors:** Maria Silvia Ferrari Lavrador, Milessa Silva Afonso, Dennys Esper Cintra, Marcia Koike, Valeria Sutti Nunes, Marina Demasi, Chin Jia Lin, Lis Mie Masuzawa Beda, Luiz Antonio Gioielli, Renata de Paula Assis Bombo, Roberta Marcondes Machado, Sergio Catanozi, Edna Regina Nakandakare, Ana Maria Lottenberg

**Affiliations:** 1 Laboratorio de Lipides (LIM10), Hospital das Clinicas HCFMUSP, Faculdade de Medicina, Universidade de Sao Paulo, Sao Paulo, SP, BR 01246-903, Brazil; mariasilviaferrari@yahoo.com.br (M.S.F.L.); milessafonso@gmail.com (M.S.A.); valeriasutti@gmail.com (V.S.N.); marinalgdemasi@gmail.com (M.D.); lismiem@yahoo.com.br (L.M.M.B.); rebombo@comcast.net (R.d.P.A.B.); rmarcondesmachado@yahoo.com.br (R.M.M.); catanozi@usp.br (S.C.); enakonda@usp.br (E.R.N.); 2 Laboratory of Nutritional Genomics-School of Applied Science, University of Campinas (UNICAMP), Limeira, São Paulo 13484-350, Brazil; dcintra@yahoo.com; 3 Emergency Care Research Unit Laboratory (LIM51), Faculty of Medical Sciences of the University of São Paulo, São Paulo 01246-903, Brazil; mkkoike17@gmail.com; 4 Laboratory of Molecular Biology (LIM22), Department of Pathology, Faculty of Medical Sciences of the University of São Paulo, São Paulo 01246-903, Brazil; cjlin@usp.br; 5 Department of Biochemical and Pharmaceutical Technology, Faculty of Pharmaceutical Sciences of the University of São Paulo, São Paulo 05508-000, Brazil; lagio@usp.br; 6 Faculdade Israelita de Ciências da Saúde Albert Einstein, São Paulo, SP, BR 05521-200, Brazil

**Keywords:** NASH, obesity, saturated fatty acid, inflammation, interesterified fat, lipids metabolism

## Abstract

Interesterified fats are being widely used by the food industry in an attempt to replace trans fatty acids. The effect of interesterified fats containing palmitic or stearic acids on lipid metabolism and inflammatory signaling pathways in adipose and hepatic tissues was evaluated. Male LDLr-KO mice were fed a high-fat diet containing polyunsaturated (PUFA), palmitic (PALM), palmitic interesterified (PALM INTER), stearic (STEAR), or stearic interesterified (STEAR INTER) fats for 16 weeks. The expression of genes and protein levels involved in lipid metabolism and inflammatory processes in liver and white adipose tissue was determined by quantitative RT-PCR and by Western blot, respectively. The infiltration of inflammatory cells in hepatic and adipose tissues was determined by eosin and hematoxylin, while liver collagen content was determined by Sirius Red staining. Both interesterified fats increased liver collagen content and JNK phosphorylation. Additionally, the STEAR INTER group developed nonalcoholic steatohepatitis (NASH) associated with higher neutrophil infiltration. PALM INTER induced adipose tissue expansion and enlargement of adipocytes. Furthermore, PALM INTER triggered increased IKK phosphorylation and TNFα protein content, conditions associated with the upstream activation of the NFkB signaling pathway. STEAR INTER induced NASH, while PALM INTER triggered hepatic fibrosis and adipocyte hypertrophy with inflammatory response in LDLr-KO mice.

## 1. Introduction

The adipose tissue (AT) is a central metabolic organ involved in the regulation of whole-body energy homeostasis [1]. The expansion of AT, notably the visceral fat depot, relates to obesity-associated metabolic complications, such as type 2 diabetes and cardiovascular disease [2]. Furthermore, dysfunctional adipocytes are potential sources of lipids to the liver, as most hepatic triglycerides are not assembled from de novo lipogenesis, but from the re-esterification of adipose-tissue-derived fatty acids [3,4]. Moreover, dietary fatty acids can modulate the cross-talk between adipocytes, liver, and skeletal muscle [5], presenting an important role in metabolic-related diseases.

Saturated fatty acids (SAFAs) modulate important signaling pathways involved in the onset and progression of obesity [6]. It has already been shown that SAFA can act as a paracrine mediator of inflammation in the interaction between adipocytes and macrophages [7]. Palmitic acid is the most abundant SAFA in the diet [8], and it contributes to the activation of inflammatory signaling pathways that impair insulin sensitivity [9] and induce adipocytes hypertrophy via macrophage-induced lipolysis [7]. Furthermore, stearic acid induces TLR4/2-independent inflammation leading to endoplasmic reticulum (ER) stress-mediated apoptosis in macrophages [10]. SAFAs also relate to liver lipotoxicity since they lead to ER stress, affecting mitochondrial metabolism, promoting the accumulation of reactive oxygen species (ROS), and also inducing apoptosis which is dependent on JNK activation [11]. 

The role of SAFAs in chronic diseases is also related to their ability to promote alterations in lipoprotein metabolism. In this regard, it was demonstrated that the enrichment of palmitic acid in hepatocytes decreases LDL receptor expression and activity, which contributes to increased LDL plasma concentrations [12,13]. On the other hand, stearic acid, the second most abundant SAFA in the diet, presents a neutral effect on plasma lipids, due to its conversion into oleic acid by stearoyl CoA desaturase (SCD-1) [14]. In the liver, SAFAs trigger hepatic lipogenesis by activating transcription factors [15] that contribute to nonalcoholic fatty liver disease (NAFLD), insulin resistance, and atherosclerosis development [16,17].

Blends of fats rich in palmitic and stearic acids are widely used to produce interesterified fats in order to meet the recommendations of health organizations to replace deleterious trans fatty acids in industrialized foods [18,19]. Interesterified fats are prepared with a mixture of vegetable oils and totally hydrogenated fat submitted to catalytic reactions that culminate in the enrichment of SAFAs in the sn-2 position of the glycerol backbone after a random rearrangement of fatty acids [20]. It is important to note that the sn-2 position is normally occupied by unsaturated fatty acids in vegetable oils [21,22]. As pancreatic lipases hydrolyze fatty acids present in the sn-1 and sn-3 positions of triglycerides [23,24], fatty acids in the sn-2 position will not be available for further desaturation in enterocytes [25,26]. Furthermore, although the expression of monoacylglycerol lipase is observed in human and rodent intestinal mucosa, it has minimal lipolytic activity [27]. For this reason, the composition of triglycerides incorporated into chylomicrons are similar to those from the dietary fat as demonstrated in humans [28]. Therefore, under the consumption of interesterified fats, greater amounts of SAFA in the sn-2 position are incorporated in both hepatic and adipose tissues [29], which may impair the cholesterol and triglycerides (TG) metabolism. However, the studies have shown a lot of controversies in this subject. Sanders and colleagues have shown that the consumption of interesterified fats reduced the postprandial lipemic response in healthy subjects [30], an effect also verified in adults with elevated plasma triglyceride concentrations [31]. On the other hand, Zock et al. (1995) did not observe any alterations in fasting plasma TG concentrations in individuals consuming interesterified palm oil for 3 weeks as compared to subjects consuming palm oil [21]. Most importantly, there is no clarification regarding the chronic effects of interesterified fat consumption on plasma lipemia. At the same time, it is not known whether the deleterious effects of saturated fatty acids in the liver and adipose tissue could be exacerbated when they are mostly present in the sn-2 position of triglycerides. Therefore, the aim of this investigation was to evaluate the chronic effect of interesterified fats containing stearic or palmitic acids on molecular signaling pathways involved in hepatic and adipose tissue lipid metabolism and inflammation in LDLr-KO mice.

## 2. Material and Methods

### 2.1. Mouse Model and Diet

Male LDLr-KO mice (C57BL/6J background) were purchased from Jackson Laboratory (Bar Harbor, USA) and housed in a conventional animal facility (12 h light/dark cycle) in a temperature-controlled environment and ad libitum food and water. For each group, 18 animals were enrolled which were recognized as appropriate models to evaluate the effects of different diets on metabolic syndrome, fatty liver disease, obesity, and diabetes [32,33,34], since they develop similar lipid profiles when compared to humans after a high-fat diet [35]. Furthermore, this animal model presents the absorption of fatty acids at the sn-2 position similar to that observed for humans [32,33]. All the animal protocols were approved by the Ethics Committee of the Faculty of Medical Sciences of the University of São Paulo (CEP 026/12) and were conducted in agreement with NIH guidelines for the care and use of laboratory animals. After weaning, mice were randomly divided into five groups and fed on high-fat diets (40% of energy as fat) enriched with polyunsaturated (PUFA), palmitic (PALM), palmitic interesterified (PALM INTER), stearic (STEAR), or stearic interesterified (STEAR INTER) fats for 16 weeks. The composition of the diets was compliant with the recommendations of the AIN [36] without cholesterol addition and had similar amounts of fiber. The fatty acid composition of the fats used in the diets was determined by gas liquid chromatography analyses (GLC) and the position of the fatty acids on the glycerol backbone was shown by nuclear magnetic resonance (NMR) employing ^13^C [37,38] (Table 1). All fats were provided by Cargill^®^ (São Paulo, SP, Brazil). Mice were weighed weekly and food intake was estimated as the difference between the food offered and the residual food in the cages. After a 16-week period, mice were fasted overnight for a period of 9 to 12 h and euthanasia was performed using a CO_2_ chamber with a gradual fill method (displacement rate from 10% to 30% of the chamber volume/min). Blood was drawn from the abdominal aorta into 0.1% EDTA tubes. After perfusion with phosphate-buffered saline (PBS), liver and epididymal and subcutaneous fat pads were weighed, frozen in liquid nitrogen, and stored at −80 °C.

### 2.2. Blood Sampling

Glucose and alanine aminotransferase (ALT) analyses were carried out using enzymatic-colorimetric kits (Labtest Diagnóstica, Vista Alegre, Lagoa Santa, MG, Brazil). Insulin concentration was measured using ELISA kit (Alpco Diagnostics, Salem, NH, USA).

### 2.3. Liver Lipid Concentrations

Measurement of liver cholesterol and TG was performed as previously described [39]. Briefly, ∼200 mg of tissue was homogenized and lipids were extracted in a chloroform:methanol (2:1) solution (6 mL). The organic phase was separated from aqueous phase by the addition of 0.05% H_2_SO_4_. Chloroform containing Triton X-100 (0.5% Triton X-100, *v*/*v*) was then added to the lower phase and dried under nitrogen. The tubes were rinsed with chloroform and dried again. Deionized water (1 mL) was added and the samples were incubated at 37 °C for 15 min under shaking. After this period, the samples were vortexed and subsequently used for the enzymatic determination of lipid content.

### 2.4. Histological Analysis

Hepatic tissue and epididymal adipose tissue fragments were fixed in 4% formaldehyde, dehydrated with ethanol, cleared with xylene, and embedded in paraffin. After inclusion, 4 μm sections were obtained and stained with hematoxylin and eosin or Picrosirius red. Photomicrographs were obtained by digital imaging system (Leica Qwin, Leica System, Cambridge, CB5 8PB, UK). The mean area of adipocytes (average surface area of 70–100 randomly sorted adipocytes per animal) was determined and quantified in Image J (Media Cybernetics, MD, USA). 

### 2.5. Grading of the NAFLD Activity

Histological variables were blindly scored by one of the authors using a scoring system adapted from Kleiner et al. [40]. This system numerically rates macrosteatosis (0–3), lobular inflammatory changes (0–3), and hepatocyte ballooning (0–2) and fibrosis (0–2). Score of 5 or more was characterized as NASH; values between 3 and 4 were allocated in the categories of absence, presence, or probable NASH; and values below 3 indicated absence of NASH. The collagen volume (%) was expressed as the percentage of area of the centrilobular vein (CLV) and portal triad using a digital image system (Qwin image analysis software; Leica Imaging Systems, Cambridge, CB5 8PB, UK).

### 2.6. Neutrophil Infiltration

HE-stained slides were evaluated under a light microscope at 400× magnification. Neutrophils were identified by nuclear and cytoplasmic appearance and counted in 20 fields of the hepatic parenchyma (3.4 mm^2^/blade) [41]. Neutrophils of central or periportal regions as well as the present inside granulomas were not considered. The inflammatory infiltrate of neutrophils in the liver parenchyma is presented as number of cells/mm^2^.

### 2.7. Immunofluorescence Analyses of M1-like Macrophages

The localization of integrin-αM was detected using anti-integrin-αM antibody (CD11b^+^, rabbit polyclonal, Santa Cruz Biotechnology, code sc28664; 1:100) by indirect immunofluorescence staining, as previously described [42]. In short, sections were fixed in acetone and blocked with 2% rabbit serum for 30 min at room temperature, followed by incubation with primary antibody anti-integrin-αM (1:200) for 12 h, at 4 °C, in a moisture chamber. Sections incubated with the anti-F4/80 antibody were then incubated with an FITC-conjugated anti-rabbit IgG secondary antibody. FITC-conjugated mice anti-rabbit antibody was from Santa Cruz Biotechnology (Santa Cruz, CA, USA). The sections were visualized under a fluorescence microscope (Leica FW 4500 B). 

### 2.8. Protein Analysis by Immunoblotting

~200 mg of liver were homogenized in extraction buffer (1% Triton-X 100, 100 mM Tris, pH 7.4, containing 100 mM sodium pyrophosphate, 100 mM sodium fluoride, 10 mM EDTA, 10 mM sodium vanadate, 2 mM PMSF, and 0.1 mg of aprotinin/mL) at 4 °C with Polytron MR 2100 (Kinematica, Luzernerstrasse, Luzern, Switzerland). The extracts were centrifuged at 11,000 rpm and 4 °C in an Eppendorf centrifuge 5804R (Eppendorf AG, Hamburg, Germany) for 40 min to remove insoluble material. The protein concentration was determined by the Biuret method [43]. Samples containing 125 μg of protein extracts (liver) were separated by SDS-PAGE, transferred to nitrocellulose membranes, and blotted with antibodies against tumor necrosis factor-alpha (TNF-α; BioLegend, code 506,102; 1:1000), interleukin-1β (IL-1β; BioLegend, code 503,502; 1:1000), phospho-c-Jun N-terminal kinase (p-JNK; Santa Cruz Biotech, code sc20140; 1:1000), phospho-inhibitor of kappa B-α (pIkB-α; Cell Signalling; code 92461:1000), phospho-IkB kinase β (p-Ikk β; Santa Cruz Biotech, code sc23,470; 1:1000), fatty acid syntase (FAS; Santa Cruz Biotech, code sc6254; 1:1000), B-cell lymphoma 2 (BCL-2; Santa Cruz Biotech, code sc783; 1:1000), Bcl-2-associated X protein (BAX; Santa Cruz Biotech, code sc6236; 1:1000), Caspase-3 (Santa Cruz Biotech, code sc7148; 1:1000), and β-actin (Santa Cruz Biotech, code sc7210; 1:5000). Chemiluminescent detection was performed with horseradish peroxidase-conjugate secondary antibodies. Specific bands were labeled by chemiluminescence and visualization was performed with a system for imaging fluorescence (G:BOX Chemi XRQ–Syngene, Frederick, MD, USA). The band intensities were quantified by optical densitometry (UN-Scan-it Gel 6.1, Orem, UT, USA) and normalized by β-actin. 

### 2.9. mRNA Analysis by RT-qPCR

RNA was isolated by Trizol/chloroform extraction and purified using the RNeasy kit (Qiagen, Toronto, ON, Canada). RNA quantity and quality were assessed using Bioanalyser (Bio-Rad, Mississauga, ON, Canada) and cDNA was prepared using High Capacity cDNA Reverse Transcription kit (Applied Biosystems, Forest City, CA, USA). Real-time PCR analysis was performed using a StepOne Plus system (Applied Biosystems). Data were analyzed using the ΔΔCT method to determine the expression level of each gene normalized to the expression level of the housekeeping HPRT and B2M for liver and adipose tissue, respectively. Primer sequences were as follows: interleukin 10 (IL10; Mm00439614_m1, *Il10*), IL1β (Mm00434228_m1, *Il1b*), interleukin 6 (IL6; Mm00550338_m1, *Il6*), TNF-α (Mm00443258_m1, *Tnf*), microsomal triglyceride transfer protein (MTTP; Mm00435015_m1, *Mttp*), sterol regulatory element-binding protein 1c (SREBP-1c; Mm00446190_m1, *Srebf1*), peroxisome proliferator-activated receptor-γ (PPARγ; Mm00435015_m1, *Pparg*), PPARα (Mm00440939_m1, *Ppara*), FAS (Mm00662319_m1, *Fasn*), carnitine palmitoyl transferase 1A (CPT-1a (Mm01231183_m1, *Cpt1a*), SCD-1 (Mm00772290_m1, *Scd1*), PGC1α(Mm00447183_m1, Ppargc1a), hormone-sensitive lipase (HSL; Mm00495359_m1, *Lipe*), adipose triglyceride lipase (ATGL; Mm00503040_m1, *Pnpla2*), Perilipin1 (Mm00558672_m1, *Plin1*), adiponectin (Mm00456425_m1, *Acrp30*), hypoxanthine-guanine phosphoribosyltransferase (HPRT; Mm03024075_m1, *Hprt*), β-2m(Mm00437762_m1, *B2m*).

### 2.10. Statistical Analyses

Analyses were carried out on GraphPad Prism software (GraphPad Software Inc., San Diego, CA, USA). All data were checked for normality prior to statistical analysis. Comparisons were made utilizing one-way ANOVA followed by the post hoc test Newman–Keuls Multiple Comparison Test for parametric and Kruskal–Wallis for nonparametric comparisons. A value of *p* < 0.05 was considered statistically significant. The post hoc test was only applied after a difference between groups.

## 3. Results

To investigate the impact of interesterified fats on adipose tissue and hepatic lipid metabolism, weaning LDLr-KO mice were randomly distributed into five groups receiving high-fat diets (40% of energy as fat). These animals develop obesity and comorbidities to a similar extent as humans submitted to a high-fat diet [32,33].

**Figure 1:**
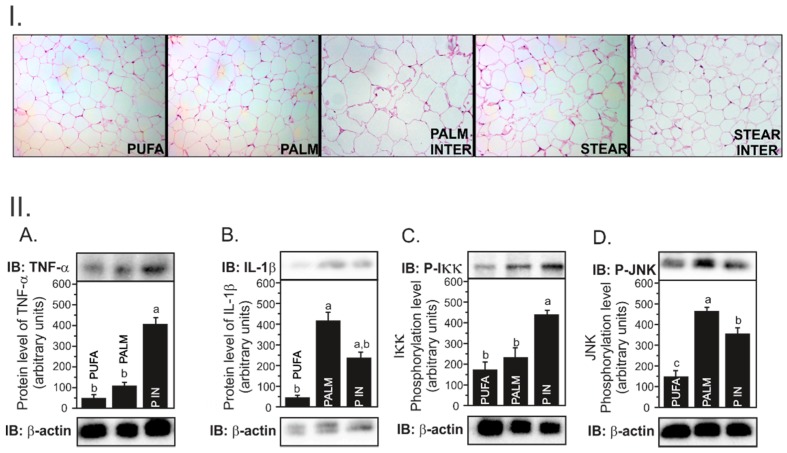
PALM INTER group promotes adipocytes hypertrophy (**I**) and induces inflammatory cytokine expression (**A**,**C**). (**I**). Photomicrography of epididymal visceral adipose tissue dissected from LDLr-KO mice fed a high-fat diet enriched with polyunsaturated fatty acids (PUFA), palmitic acid (PALM), palmitic interesterified fat (PALM INTER), stearic acid (STEAR), and stearic interesterified fat (STEAR INTER) for 16 weeks. Sections were stained with hematoxylin eosin and photomicrography is presented under 20x objective lens. (**II**). Western blot analysis from Both stearic groups (STEAR and STEAR INTER groups) was not performed (**A**–**D**) because of the small amount of adipose tissue obtained in these groups. Data were checked for normality prior to statistical analysis. One-way ANOVA was performed, followed by the post hoc Newman–Keuls test. Statistically significant difference is presented by different letters (*p* < 0.05) with a > b > c values. Data are shown as mean ± SEM (*n* = 6).

All animals presented the same body weight at baseline (*p* = 0.76) and dietary intake (*p* = 0.15) did not differ among the groups. After 16 weeks, PALM INTER presented increased body weight as compared to STEAR and STEAR INTER (*p* < 0.01), but not when compared to PUFA and PALM groups. Concurrently, PALM INTER had greater visceral (*p* < 0.001) and subcutaneous (*p* < 0.001) fat contents and epididymal adipocyte diameter (*p* < 0.001) (Figure 1(I)) when compared to the other groups (Table 2). 

Glucose and insulin concentrations were not different between PALM and PALM INTER groups nor between STEAR and STEAR INTER groups, meaning that the interesterification process did not alter these parameters (Table 3). However, glucose and insulin concentrations were significantly higher for PALM and PALM INTER (*p* < 0.05) as compared to the other treatments.

PALM INTER induces inflammation. The adipocyte hypertrophy observed in the PALM INTER group led us to investigate the expression of genes related to lipolysis and inflammatory response. PALM INTER-fed mice had reduced expression of ATGL (*Pnpla2*) as compared to PALM, but no differences were observed regarding other lipid droplet-associated protein, such as perilipin (*Plin*, *p* = 0.34) and hormone-sensitive lipase (*Lipe*, *p* = 0.61) (Table 4). A higher *Scd1* expression was observed in STEAR- and STEAR INTER-fed mice as compared to PUFA (*p* < 0.05), since stearic acid is the main substrate for this enzyme. There were also no differences in the expression of genes involved in the inflammatory response, such as adiponectin (*Acrp30*, *p* = 0.07) and TNFα (*Tnf*, *p* = 0.65).

Although we did not find any difference in cytokines gene expression among the groups, PALM INTER-fed mice presented increased TNFα (*p* < 0.001) and pIKK (*p* < 0.05) protein levels (Figure 1(IIA,C), respectively). However, IL1β and pJNK levels were not different among groups, which suggests that PALM INTER induced TNFα protein concentration in adipose tissue through the activation of the upstream NFkB inflammatory signaling pathway. 

**Figure 2:**
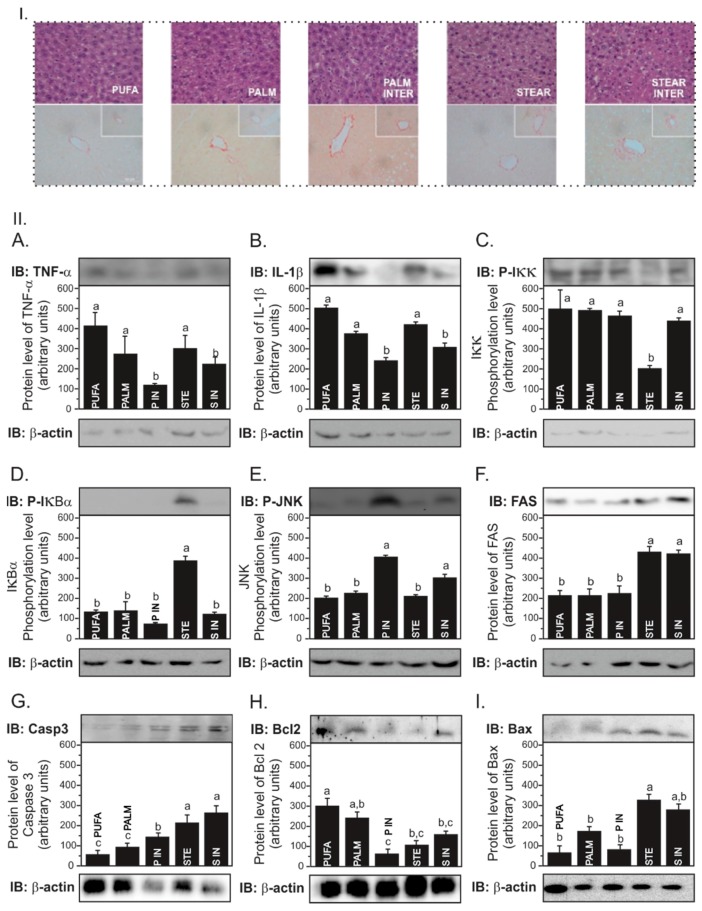

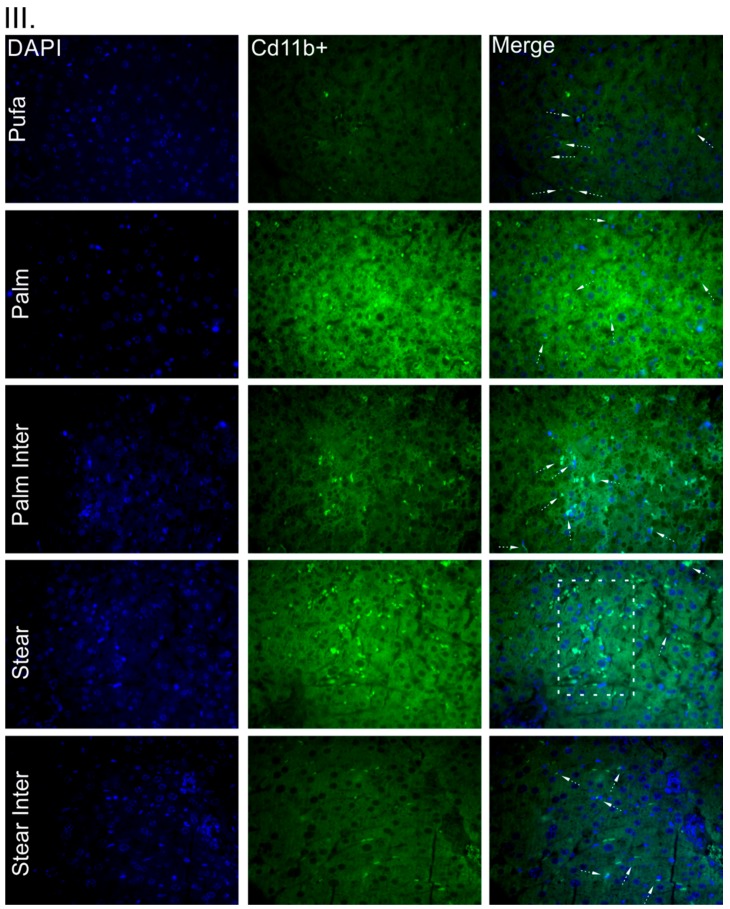
Interesterified fats induce higher collagen content, but STEAR INTER group presents NASH (score = 5). Section **I**. Photomicrography of hepatic tissue dissected from LDLr-KO mice fed a high-fat diet enriched with polyunsaturated fatty acids (PUFA), palmitic acid (PALM), palmitic interesterified fat (PALM INTER), stearic acid (STEAR), and stearic interesterified fat (STEAR INTER). Sections were stained with Hematoxylin Eosin (HE) for neutrophil content (identified by nuclear and cytoplasmic appearance and counted in 20 fields of the hepatic parenchyma (3.4 mm^2^/blade) and stained with Picrosirius red for collagen content (volume %) and expressed as the percentage of area of the centrilobular vein and portal triad) (*n* = 10). Photomicrography of HE and Picrosirius red are under 20× and 40× objective lens, respectively. Liver disease scores: PUFA and STEAR = 2; PALM and PALM INTER = 4; STEAR INTER = 5. M1-like macrophages in the hepatic tissue of LDLr-KO mice. Representative photomicrography. Section **II**. (**A**–**I**) Data were checked for normality prior to statistical analysis. One-way ANOVA was performed, followed by the post hoc Newman–Keuls test. Statistically significant difference is presented by different letters (*p* < 0.05) with a > b > c values. Data are shown as mean ± SEM (*n* = 6). Section **III**. White arrows or white-segmented board indicating the presence of CD11b+ cells in liver sections from mice fed five of the different fats are presented under 200× magnification.

STEAR INTER induces NASH and both interesterified fats increase collagen content in the liver. Hepatic lipid infiltration was observed in all groups. Liver weight (*p* = 0.66), cholesterol (*p* = 0.06), and TG (*p* = 0.78) concentrations were not significantly different among groups (Table 3). STEAR INTER-fed mice developed NASH-like lesions characterized by intermediate steatosis (grade 2), inflammatory infiltrate (grade 1), and fibrosis in both centrilobular and portal triad veins (grade 2), whereas PALM INTER- and PALM-fed mice had mild intermediate steatosis (grade 2) with minimal inflammatory process (grade 1) and fibrosis (grade 1) (Figure 2(I)). Hepatocellular ballooning was not observed in all samples and was therefore not scored. STEAR INTER induced more neutrophil infiltration (*p* < 0.001) compared to the others and higher collagen content in the centrilobular vein compared to STEAR and PUFA (*p* < 0.05). On the other hand, the PALM INTER group presented higher collagen content in the centrilobular (*p* < 0.05) and portal triad (*p* < 0.05) veins as compared to PALM and PUFA groups (Table 3; Figure 2(I)).

Interesterified fats induce JNK activity and PALM INTER triggers apoptosis. In order to investigate the molecular mechanisms underlying NASH development and the presence of fibrosis, the expression of genes related to hepatic lipid metabolism and inflammatory response was determined. The expression of *Scd1* was threefold and twofold greater in PALM INTER-fed mice as compared to PUFA- and PALM-fed mice (*p* < 0.05), respectively (Table 5). Although STEAR INTER induced a twofold expression of *Scd1* as compared to STEAR and PUFA, the difference was not significant (*p* = 0.11). There was also no difference in the expression of genes related to fatty acid oxidation (*Ppar-a* and *Cpt-1*), inflammation (*Il-1b*, *Il-6*, and *Tnf*), and lipogenesis (*Srebp-1c*, *Pparg* and *Mttp*).

Both interesterified fats increased JNK phosphorylation (*p* < 0.001; Figure 2(IIE)) and presented lower TNF-α (Figure 2(IIA)) and IL-1β (*p* < 0.001; Figure 2(IIB)) level compared to other fats. In addition, PALM INTER- and STEAR INTER-fed mice had less infiltration of M1-like macrophages in the liver, as shown by CD11b staining, which corroborates the immunoblotting results (Figure 2(III)). Regarding the apoptotic signaling, both STEAR and STEAR INTER showed higher caspase 3 protein levels (*p* < 0.001) than other experimental diets. Interestingly, PALM INTER presented a higher caspase-3 expression as compared to PALM (*p* < 0.01; Figure 2(IIG)). Corroborating this data, the antiapoptotic protein Bcl2 was reduced in PALM INTER (*p* < 0.01) as compared to PALM and PUFA (Figure 2(IIH)). 

## 4. Discussion

The strength of our investigation was that the interesterification process and the enrichment of palmitic acid on the sn-2 position of triglycerides elicits harmful effects on hepatic and adipose tissues in LDLr-KO mice, triggering the onset of obesity.

Since glucose and insulin plasma concentrations are also influenced by dietary fats [44], we hypothesized that the change of the position of fatty acids in the glycerol backbone could also interfere in these parameters. We observed an increase in glucose and insulin concentrations in PALM and PALM INTER as compared to the other groups. Accordingly, long-term exposure to palmitate culminates in beta cell death [45] and impaired glucose-induced generation of the second messenger cAMP, an important determinant of insulin secretion [46]. Furthermore, chronic exposure to palmitate promotes ceramide synthesis [45], which contributes to Akt inhibition and impairment in insulin sensitivity [47]. Although in our investigation SAFA impaired glucose and insulin concentration, no differences were observed between PALM and PALM INTER or between STEAR and STEAR INTER. Therefore, the set of these initial results shows that the interesterification did not alter biochemical parameters. Additionally, our previous study [48] showed that the interesterification process did not influence total plasma cholesterol, although it promoted cholesterol accumulation in LDL particles.

To the best of our knowledge, there are no investigations regarding the influence of interesterified fats on adipose tissue metabolism, as well as in the adipocytes inflammatory signaling pathways. Obesity is characterized by adipose tissue expansion with large adipocytes [49], proinflammatory cell infiltration, and subsequent secretion of inflammatory biomarkers [50]. Altogether, these conditions contribute to an impairment in insulin sensitivity [51] and higher lipolysis [52] in adipose tissue. An important finding of our investigation was that PALM INTER induced greater adipose tissue expansion and adipocytes hypertrophy. 

Therefore, our next goal was to elucidate the underlying molecular mechanisms involved in adipocyte hypertrophy. It has already been demonstrated in 3T3-L1 cells that palmitic acid increases the expression and secretion of inflammatory cytokines, such as IL-6 and TNF-α, as well as NF-κB activity in adipocytes [53]. When comparing the effects of different fatty acids on the expression of inflammatory cytokines, no differences were observed among the groups. In the case of the PUFA group, the inflammation was observed presumably because of the high concentration of omega-6 (18:2n-6), as also shown by others [54]. Arachidonic acid, the main product of 18:2n-6, is a substrate for eicosanoid synthesis which is enrolled in the control of intensity and duration of inflammatory responses [55]. However, PALM INTER presented higher TNF-α protein levels in comparison to PALM and PUFA, and one possible explanation is the higher incorporation of palmitic acid in adipocyte phospholipids, as observed in a previous study conducted in rats [29]. The adipocyte hypertrophy and TNF-α secretion are the most relevant contributors to the development of insulin resistance in adipose tissue [47]. TNFα stimulates lipolysis through attenuation of the insulin antilipolytic activity [6], increasing the release of free fatty acids, which are the major determinants of hepatic insulin resistance (IR) and lipogenesis, which culminates in NAFLD [49,50,52]. The hypertrophy of adipose tissue promotes the release of inflammatory cytokines leading to the overexpression of MCP-1, which supports the recruitment of macrophages to the adipose tissue [49]. Moreover, macrophages within expanded adipose tissue can secrete TNFα through the IKKβ–NFκB or JNK–AP1 (Jun N-terminal kinase-mitogen-activator protein-1) signaling pathway [6]. Both IKKβ and JNK are serine kinases that disrupt insulin signaling pathways, eliciting adipose tissue inflammation [51]. In our investigation, PALM INTER presented the highest phosphorylation of IKKα/β. It is well documented that the IKKβ–NF-κB signaling pathway not only induces the transcription of proinflammatory mediators, such as IL-1β, IL-6, TNF-α, but also contributes to IR and NAFLD development [52].

Regarding the effects of the interesterified fats on obesity development, a study conducted on mice showed that the maternal intake of palmitic or interesterified fat enriched with palmitic acid during pregnancy and lactation predisposed the offspring to the development of obesity in adult life, which was attributed to interesterified fats adversely influencing epigenetics [53]. According to another study, a higher concentration of palmitic acid was incorporated into adipocyte phospholipids of rats submitted to a diet enriched with palmitic acid in the sn-2 position of the glycerol backbone [29].

We were also looking at identifying the impact of interesterified fats on hepatic lipid metabolism, since a high-fat diet [56], particularly high in saturated fat, is related to hepatic IR and TG accumulation [6], promoting inflammation and fibrosis [57]. We did not find differences in triglyceride content among groups, since all animals developed NAFLD because of the high-fat diet. Similar results were found by Ebbert & Jensen [58] in the same animal model submitted to a high-SAFA or high-PUFA diet.

Although PALM INTER presented a less inflammatory cytokine level (TNF-α and IL-1β), this group showed higher fibrosis and apoptosis as compared to PALM and PUFA, as well as higher JNK activation. Fibrosis and apoptosis processes are late events in NAFLD [59]. Furthermore, although inflammation classically precedes fibrosis, the amount of fibrosis is not automatically associated with the severity of inflammation, indicating that the mechanisms that regulate fibrogenesis may be different from those that regulate inflammation [60]. Thus, fibrosis is not permanently characterized by persistent inflammation that usually occurs after higher inflammation degree and may explain lower levels of TNF-α and IL-1β. JNK is a serine kinase involved in the inflammatory signaling pathway that also activates apoptotic proteins, such as Bax. It was demonstrated in vitro that hepatocytes treated with monounsaturated and saturated fatty acids induced the same degree of steatosis, higher apoptosis, related to JNK activation [61].

Moreover, STEAR INTER-fed mice presented higher pJNK without expressing apoptosis markers, but this was the only group presenting NASH-like lesions with higher neutrophils infiltration as compared to all groups, probably because of the interesterification process, since the amount of SAFAs in the sn-2 position remained the same. However, STEAR INTER-fed mice did not present higher adipose tissue expansion as compared to the others. In fact, it was already demonstrated that among SAFAs, stearic acid has a higher melting point, which hinders micelle formation, contributing to less absorption and adipose tissue formation in animals fed with these fats [62]. 

Altogether, our results showed that interesterified fats enriched with palmitic or stearic acids induced hepatic fibrosis, whereas PALM INTER triggered adipocytes hypertrophy in LDLr-KO mice. As interesterified fats are largely utilized by the food industry, the adverse effects shown here warrant further investigation in humans.

## 5. Limitations of the Study

The limitations of this study refer to the NMR analysis lacking the ability to determine the position of fatty acids in the glycerol backbone from triglycerides extracted from liver and adipose tissue. We were also not able to determine the composition of chylomicrons due to the complexity of the method when realized in mice.

## Figures and Tables

**Table 1:** Fatty acid composition of fats (percentage of total fat) and regiospecific distribution of fatty acids in sn-1,3 and sn-2 positions in triacylglycerols of PALM, PALM INTER, STEAR, and STEAR INTER fats.

Fatty Acids	PUFA	PALM	PALM INTER	STEAR	STEAR INTER
Lauric 12:0	0	0.24	0.22	0.06	0.07
Miristic 14:0	0.07	0.92	0.95	0.11	0.08
Palmitic 16:0	5.49	40.00	40.82	5.82	5.02
Stearic 18:0	3.17	5.06	4.60	39.72	40.47
Elaidic 18:1t	0.05	0.22	0.08	0.26	0.07
Oleic 18:1c	39.94	39.89	39.68	40.35	40.38
Linoelaidic 18:2t	0.25	0.72	0.39	0.27	0.12
Linoleic 18:2n-6	45.55	11.33	11.70	9.79	10.16
Linolenic 18:3n-3	3.12	0.30	0.41	0.88	1.26
Araquidic 20:0	0.47	0.43	0.39	1.05	1.06
Eicosenoic 20:1	0.72	0.29	0.22	0.46	0.34
Docosanoic 22:0	0.68	0.11	0.10	0.71	0.68
Others	0.49	0.49	0.44	0.52	0.29
Total SAFA	9.88	46.76	47.08	47.47	47.38
Total MUFA	40.66	40.18	39.9	40.81	40.72
Total PUFA	48.67	11.63	12.11	10.67	11.42
Total TRANS	0.44	1.04	0.55	0.73	0.25
Fatty acids (% position)					
SAFA (sn-1,3)		67.7	50.4	56.2	48.9
SAFA (sn-2)		9.7	49.0	47.3	45.3
MUFA (sn-1,3)		25.2	37.0	34.2	40.2
MUFA (sn-2)		65.1	37.5	36.8	41.4
PUFA (sn-1,3)		7.1	12.7	9.6	10.9
PUFA (sn-2)		25.2	13.5	15.9	15.4

Sources of fats: PUFA (60% sunflower oil, 40% canola oil); PALM (95% palm oil, 4% soybean oil, 1% canola oil); PALM INTER (95% palm oil, 4% soybean oil, 1% canola oil); STEAR (42% totally hydrogenated canola oil, 40.5% high-oleic sunflower oil, 2% soybean oil); STEAR INTER (42% totally hydrogenated canola oil, 40.5% high-oleic sunflower oil, 2% soybean oil). Fats were generously provided by Cargill, SP, Brazil.

**Table 2:** Initial weight, food intake, body weight gain, relative weight of adipose tissue, adipocyte area of LDLr-KO mice fed experimental diets for 16 weeks.

	PUFA	PALM	PALM INTER	STEAR	STEAR INTER
Initial weight (g)	13.5 ± 2.6	13.5 ± 3.2	13.7 ± 3.3	13.7 ± 2.7	14.6 ± 2.7
Food intake (g/d/animal)	2.9 ± 0.3	3.2 ± 0.8	3.2 ± 0.4	3.5 ± 0.5	3.3 ± 0.2
-Weight gain (g)	16.0 ± 3.1 ^a,b^	16.1 ± 2.9 ^a,b^	18.1 ± 4.2 ^a^	14.1 ± 2.7 ^b^	14.3 ± 3.0 ^b^
Visceral AT (g/100g of body weight)	3.2 ± 0.8 ^b^	3.3 ± 0.8 ^b^	4.0 ± 0.5 ^a^	2.0 ± 0.6 ^c^	2.8 ± 0.9 ^b^
Adipocyte area (μm^2^)	3101 ± 648 ^b^	2804 ± 703 ^b^	4356 ± 1190 ^a^	2821 ± 604 ^b^	2110 ± 714 ^b^
Subcutaneous AT (g/100g of body weight)	1.7 ± 0.4 ^b^	1.6 ± 0.5 ^b^	2.2 ± 0.4 ^a^	1.1 ± 0.3 ^c^	1.5 ± 0.5 ^b^

Data were checked for normality prior to statistical analysis. One-way ANOVA was performed, followed by the post hoc Newman–Keuls Multiple Comparison Test. Statistically significant differences are represented by different letters (*p* < 0.05), with a > b > c values. Data presented as mean ± SD (*n* = 18). Legend: AT = white adipose tissue, high-fat diet enriched with polyunsaturated fatty acids (PUFA), palmitic acid (PALM), palmitic interesterified fat (PALM INTER), stearic acid (STEAR), and stearic interesterified fat (STEAR INTER).

**Table 3:** Plasma parameters, relative weight of liver, cholesterol and triglycerides content, neutrophils infiltration, and collagen content in LDLr-KO mice fed experimental diets for 16 weeks.

	PUFA	PALM	PALM INTER	STEAR	STEAR INTER
ALT (mg/dL)	37.7 ± 12.4	61.3 ± 7.6	51.6 ± 12.2	48.4 ± 14.4	58.0 ± 14.1
Glucose (mg/dL)	232 ± 13.0 ^b^	336 ± 33.9 ^a^	328 ± 27.6 ^a^	211 ± 20.1 ^b^	288± 15.7 ^b^
Insulin (mg/dL)	0.3 ± 0.0 ^b^	0.8 ± 0.1 ^a^	0.7 ± 0.1 ^a^	0.3 ± 0.1 ^b^	0.2 ± 0.0 ^b^
Liver weight (g/100g body weight)	4.4 ± 0.6	4.7 ± 1.1	4.5 ± 0.6	4.5 ± 0.4	4.4 ± 0.8
Cholesterol (g/100g liver)	1.7 ± 0.3	2.1 ± 0.6	1.7± 0.4	1.7 ± 0.4	1.9 ± 0.5
Triglycerides (g/100g liver)	15.1 ± 7.7	16.7 ± 11.2	15.6 ± 5.4	16.5 ± 8.4	13.5 ± 5.4
Neutrophils infiltration (cell/mm^2^)	1.8 ± 0.8 ^b^	2.3 ± 0.9 ^b^	3.0 ± 1.9 ^b^	2.0 ± 0.8 ^b^	6.1 ± 3.9 ^a^
Collagen content CLV * (%)	24.3 ± 0.8 ^b^	14.7 ± 7.3 ^b^	34.8 ± 20.3 ^a^	15.2 ± 9.9 ^b^	31.6 ± 13 ^a^
Collagen content triad (%)	25.9 ± 9.9 ^b^	31.8 ± 14.2 ^b^	51.2 ± 9.7 ^a^	32.0 ± 14.9 ^b^	36.6 ± 18.6 ^b^

Liver samples were dissected from LDLr-KO mice fed a high-fat diet enriched with polyunsaturated fatty acids (PUFA), palmitic acid (PALM), palmitic interesterified fat (PALM INTER), stearic acid (STEAR), and stearic interesterified fat (STEAR INTER) for 16 weeks. Data were checked for normality prior to statistical analysis. One-way ANOVA was performed, followed by the post hoc Newman–Keuls Multiple Comparison Test. ALT and glucose Kruskal–Wallis were performed, followed by post hoc Dunn. Statistically significant differences are represented by different letters (*p* < 0.05) with a > b values. Data are shown as mean ± SD (*n* = 14−19). Neutrophils were counted in 20 fields of the hepatic parenchyma under 40x magnification (*n* = 8–10). * CLV: Centrilobular vein.

**Table 4:** Adipose tissue mRNA related to inflammatory and metabolic pathways of LDLr-KO mice fed experimental diets for 16 weeks.

	PUFA	PALM	PALM INTER	STEAR	STEAR INTER
*Tnf/b2m*	1.35 ± 0.26	1.29 ± 0.14	1.10 ± 0.19	1.14 ± 0.18	0.97 ± 0.09
*Acrp30/b2m*	0.86 ± 0.08	0.74 ± 0.05	0.61 ± 0.07	0.80 ± 0.12	0.76 ± 0.08
*Plin/b2m*	0.42 ± 0.07	0.47 ± 0.04	0.39 ± 0.03	0.41 ± 0.07	0.46 ± 0.04
*Pnpla2/b2m*	0.80 ± 0.16 ^b^	1.28 ± 0.11 ^a^	0.74 ± 0.08 ^b^	1.02 ± 0.20 ^b^	0.89 ± 0.16 ^b^
*Lipe/b2m*	0.58 ± 0.12	0.70 ± 0.03	0.62 ± 0.06	0.59 ± 0.08	0.55 ± 0.06
*Scd1/b2m*	0.89 ± 0.27 ^b^	1.60 ± 0.30 ^a,b^	1.59 ± 0.83 ^a,b^	1.99 ± 0.40 ^a^	2.21 ± 0.98 ^a^

Adipose tissue samples were dissected from LDLr-KO mice fed a high-fat diet enriched with polyunsaturated fatty acids (PUFA), palmitic acid (PALM), palmitic interesterified fat (INTER PALM), stearic acid (STEAR), and stearic interesterified fat (INTER STEAR) for 16 weeks. Results were standardized to beta-2-microglobulin (*b2m*) and normalized to the expression of adipose samples from mice fed a diet containing 7% of energy as fat. Data were checked for normality prior to statistical analysis. One-way ANOVA was performed, followed by the post hoc Newman–Keuls Multiple Comparison Test. Statistically significant difference is represented by different letters (*p* < 0.05) with a > b values. Data presented as mean ± SD (*n* = 5–7).

**Table 5:** Liver mRNA related to inflammatory and metabolic pathways of LDLr-KO mice fed experimental diets for 16 weeks.

	PUFA	PALM	PALM INTER	STEAR	STEAR INTER
*Tnf/Hprt*	0.37 ± 0.25	0.39 ± 0.23	0.29 ± 0.19	0.30 ± 0.20	0.28 ± 0.16
*Il-1b/Hprt*	0.37 ± 0.24	0.49 ± 0.23	0.38 ± 0.21	0.38 ± 0.23	0.18 ± 0.04
*Il-6/Hprt*	0.20 ± 0.05	0.24 ± 0.05	0.16 ± 0.04	0.18 ± 0.04	0.13 ± 0.02
*Il-10/Hprt*	0.24 ± 0.07	0.24 ± 0.04	0.32 ± 0.08	0.19 ± 0.05	0.24 ± 0.06
*Scd-1/Hprt*	0.08 ± 0.03 ^b^	0.09 ± 0.02 ^b^	0.21 ± 0.05 ^a^	0.07 ± 0.01 ^b^	0.15 ± 0.02 ^a.b^
*Cpt-1/Hprt*	0.21 ± 0.02	0.22 ± 0.04	0.21 ± 0.02	0.24 ± 0.04	0.17 ± 0.02
*Mttp/Hprt*	0.11 ± 0.03	0.12 ± 0.02	0.12 ± 0.02	0.16 ± 0.02	0.11 ± 0.03
*Ppar-a/Hprt*	0.11 ± 0.03	0.07 ± 0.03	0.16 ± 0.05	0.21 ± 0.06	0.17 ± 0.06
*Srebp-1c/Hprt*	0.12 ± 0.03	0.16 ± 0.04	0.14 ± 0.05	0.19 ± 0.04	0.17 ± 0.04
*Ppar-g/Hprt*	0.14 ± 0.04	0.05 ± 0.01	0.09 ± 0.02	0.14 ± 0.03	0.17 ± 0.03

Liver samples were dissected from LDLr-KO mice fed a high-fat diet enriched with polyunsaturated fatty acids (PUFA), palmitic acid (PALM), palmitic interesterified fat (PALM INTER), stearic acid (STEAR), and stearic interesterified fat (STEAR INTER) for 16 weeks. Results were standardized to hypoxanthine guanine phosphoribosyl transferase (*Hprt*) and normalized to the expression of hepatic samples from mice fed a diet containing 7% of energy as fat. Data were checked for normality prior to statistical analysis. One-way ANOVA was performed, followed by the post hoc Newman–Keuls Multiple Comparison Test. Statistically significant difference is represented by different letters (*p* < 0.05) with a > b values. Data presented as mean ± SD (*n* = 7).

## Abbreviations

AP1	Activator protein 1
ALT	alanine amino-transferase-1
AT	adipose tissue
BCL-2	B-cell lymphoma 2
BAX	Bcl-2-associated X protein
FAS	Fatty acid synthase
IkB-α	inhibitor of kappa B-α
FAS	Fatty acid synthase
IkB-α	inhibitor of kappa B-α
IKK	inhibitor of kappa B kinase
JNK	c-Jun N-terminal kinase
LDLr-KO	LDL receptor knockout
NAFLD	nonalcoholic fatty liver disease
NASH	nonalcoholic steatohepatitis
NFkB	factor nuclear kappa B
PALM	diet enriched with palmitic acid
PALM INTER	diet enriched with palmitic acid in the interesterified form
PUFA	diet enriched with polyunsaturated fatty acids
SAFA	saturated fatty acids
STEAR	diet enriched with stearic acid
STEAR INTER	diet enriched with stearic acid in the interesterified form
TG	triglyceride
TNFα	tumor necrosis factor alpha
